# Persistent Inflammatory Activity in Blood Cells and Artery Tissue from Patients with Previous Bare Metal Stent

**DOI:** 10.5935/abc.20180142

**Published:** 2018-08

**Authors:** Francisco Antonio Helfenstein Fonseca

**Affiliations:** Universidade Federal de São Paulo (UNIFESP), São Paulo, SP – Brazil

**Keywords:** Myocardial Revascularization, Stents, Inflammation, Coronary Restenosis, Anti-Inflammatory Agents/therapy

The interesting article by Farsky et al.^1^ examined the persistence of inflammatory activity after bare metal stent implantation in patients later submitted to coronary artery bypass grafting (CABG).

The authors evidenced a higher systemic expression of the gene that encodes tumor necrosis factor-alpha (TNF-alpha) in peripheral blood cells, as well as a higher tissue expression of TNF-alpha and interleukin-6 (IL-6) in patients with previous bare metal stent implantation as compared to those of revascularized individuals without previous percutaneous stent placement.

Those authors concluded that, even several months or years after stent implantation, there were markers of persistent inflammatory activity, which could be associated with less favorable outcome of CABG.

The relationship between inflammatory markers and coronary restenosis after stent placement has been recognized for years.^2^ There are few reports on the local inflammatory characteristics expressed by tissue markers in samples obtained during CABG.

The increase in circulating IL-6 levels has been associated with the increase in coronary events. A study of Mendelian randomization involving 40 studies and 133449 patients has shown that polymorphism of the gene encoding the IL-6 receptor was related to a significant reduction in the incidence of coronary events, suggesting a causal role in atherosclerosis.^3^ Another meta-analysis of genetic data has confirmed the causal role of IL-6 in atherothrombosis.^3^ Those two studies have suggested that the IL-6-mediated inflammatory pathway, from its interaction with the receptor, is involved in cardiovascular events.

The TNF-alpha, another biomarker of higher expression evidenced in the study by Farsky et al.,^1^ seems to be implicated in atherosclerotic plaque instability.^4^

Despite the substantial advance in surgical and percutaneous procedures, as well as in the clinical therapy involving new antiplatelet, anticoagulant, lipid-lowering, anti-hypertensive and anti-hyperglycemic agents, the residual risk remains elevated and new anti-inflammatory therapies have been proposed.^5^

The CANTOS study,^6^ a prospective, randomized, placebo-controlled clinical trial, involving post-myocardial infarction patients who maintained elevated high-sensitivity C-reactive protein levels, has shown that treating inflammation with the monoclonal antibody canakinumab reduced inflammatory markers and cardiovascular events during clinical drug treatment.

Those data show the relevance of the findings of the study by Farsky et al.^1^ and suggest that patients receiving bare metal stents might need additional anti-inflammatory therapy. However, new prospective studies of efficacy and safety, in addition to lower cost of that therapy, are required to its definitive incorporation into clinical practice.^7^
